# Experiments on the Substance Vulgarly Called Gum Kino

**Published:** 1804-03-01

**Authors:** 


### Experiments *on the Substance vulgarly called* Gum Kino. *By* Cit. Vauquelin.

THE name given to this substance is by no means suited to it; and were it not a common practice to give names to things before we are acquainted with their nature, it would be inconceivable how it should have been called a gum, having neither the physical nor chemical properties of one.

Neither have we any accurate knowledge of the tree or of the country that produces it; but it appears to have been first brought to Europe by the English, who made known its medicinal properties, and introduced it into our shops.

It is called in trade *kino* or the *gum-resin* of Gambia. Dr. Oldfield, who made it known to Fothergill, termed it the true gum of Senegal. In the Medical Observations and Inquiries, it is said to be brought from Africa, and the tree that furnishes it to be called by the natives *pau de sangue*.

As a medicine it is used in the form of bolusses, lozenges, aqueous infusion, and spirituous tincture, as a tonic and astringent.

Subjected to the action of fire, it melts and swells up considerably; yields at first a clear liquor, which in a few instants become coloured; a light and nearly white oil then passes over, which in the course of the process becomes coloured and heavier than the aqueous product. A small quantity of carbonic acid is likewise formed, with a large quantity of carbonated hydrogen gas.

The oil produced in this operation unites with caustic fixed alkalis, and forms a deep red liquor, that becomes of a dull green on exposure to the air.

The aqueous product is not acid, but has an acrid burning taste, owing to a portion of the oil retained in solution; and potash separates from it a large quantity of ammoniac.

Twenty grammes distilled with a strong heat left eight and half of a very bulky coal, marked with the colours of the rainbow; and this coal afforded seventy-two centigrammes of ashes, consisting chiefly of lime, silex, alumin, and oxyd of iron.

Kino is little soluble in cold water, but much more in hot, though a portion of it is insoluble. The solution is slightly acid; alcohol does not precipitate it, but separates some reddish flocks; when made with boiling water it grows turbid on cooling, and deposits a brown red precipitate.

A saturated solution is precipitated by mild alkalis, but water in sufficient quantity re-dissolves the precipitate.

Caustic alkalis likewise precipitate it, but if added in excess re-dissolve the precipitate.

Glue dissolved in water forms a very considerable rose-coloured coagulum with the solution of kino; and if the quantities be such, that both substances are saturated, the supernatant fluid will be nearly colourless.

Though these appearances indicate the presence of tannin in kino, it does not precipitate ferruginous salts black, but of a beautiful deep green, scarcely alterable by exposure to the air. This property it has in common with the infusions of cinchona and rhubarb; whence it is probable, that these three substances contain a principle of similar nature.

This principle, whatever it be, is very destructible; for, if a little oxygenated muriatic acid be poured on the precipitate it forms with iron, this loses its colour, and does not re-appear on the addition of an alkaline carbonate, which produces only a red oxyd of iron.

The solution of kino copiously precipitates acetite of lead of a yellowish grey; nitrate of silver of a reddish yellow, and tartrite of antimony of a yellowish white, but much more copiously than the infusion of tan or of cinchona; which seems to indicate, that it would be a better antidote in cases of persons poisoned by this metallic salt.

Wool and cotton being boiled in a solution of kino, and then dipped in a bath of sulphate of iron, appeared on immersion of a bottle-green; but being washed and dried, the colour became a blackish brown. It was very durable.

Hot alcohol dissolves kino very well, all but a small portion. Water renders the solution a little turbid, but precipitates nothing.

The portion insoluble in alcohol, nearly a fourth of the whole, has neither the bitterness nor astringent taste of kino; but, on the contrary, is rather mucous and sweet. It easily dissolves in hot water, and gives it a fine red colour. It is precipitable by alcohol; but neither by glue, nor by any metallic solution. On burning, it diffuses a smell resembling that of gum.

I suspect the presence of this substance favours the solution in water of the principle soluble in alcohol; for the latter is less soluble in water when separated from the former; and if the quantity of water necessary for dissolving

 the the astringent part be not employed in the first instance, what is left requires a greater proportion of water.

Four litres of water, used at different times, left near twenty grammes out of a hundred of kino undissolved.— The residuum grew soft like a resin in boiling water, and all of it, except seven decigrammes, was soluble by alcohol, to which it imparted all the properties before observed in the astringent matter.

Sulphuric acid diminishes the action of water on kino, instead of increasing it, as it does with respect to the resinous part of cinchona.

It is capable of being used for tanning leather.

From what has been said, it appears that the greater part of kino consists of tannin, and is neither a gum, nor a gum resin. But there is a slight difference between it and the tannin of galls and oak bark, which precipitate iron of a blue black, while kino precipitates it green, in which it resembles cinchona and rhubarb. If, therefore, it were to become plentiful and cheap, it might be employed for all the purposes for which astringent vegetables are commonly used.

---

Mr. Vauquelin is not the first who discovered the common error respecting kino. In the New Edinburgh Dispensatory, Dr. Duncan has entered pretty fully into the subject, and asserts it to be in reality an extract; and that what we have now in the shops is not brought from Africa, but chiefly from Jamaica. He adds, (in a letter to Mr. Nicholson) that this is an extract of the *coccoloba urifera*, or sea-side grape; while the finest kino of the shops, and what from some circumstances he supposes was the sort analyzed by Vauquelin, is the product of different species of *eucalyptus*, particularly the *resinefera*, or brown gum tree of Botany Bay, from which country a parcel was imported some years ago.

